# A study to derive a clinical decision rule for triage of emergency department patients with chest pain: design and methodology

**DOI:** 10.1186/1471-227X-8-3

**Published:** 2008-02-06

**Authors:** Erik P Hess, George A Wells, Allan Jaffe, Ian G Stiell

**Affiliations:** 1 Department of Emergency Medicine, University of Ottawa, Ottawa, Canada; 2 Department of Epidemiology and Community Medicine, University of Ottawa, Ottawa, Canada; 3 Department of Internal Medicine, Division of Cardiology, Mayo Clinic College of Medicine, Rochester, USA; 4 Department of Emergency Medicine, Department of Epidemiology and Community Medicine, University of Ottawa, Ottawa, Canada

## Abstract

**Background:**

Chest pain is the second most common chief complaint in North American emergency departments. Data from the U.S. suggest that 2.1% of patients with acute myocardial infarction and 2.3% of patients with unstable angina are misdiagnosed, with slightly higher rates reported in a recent Canadian study (4.6% and 6.4%, respectively). Information obtained from the history, 12-lead ECG, and a single set of cardiac enzymes is unable to identify patients who are safe for early discharge with sufficient sensitivity. The 2007 ACC/AHA guidelines for UA/NSTEMI do not identify patients at low risk for adverse cardiac events who can be safely discharged without provocative testing. As a result large numbers of low risk patients are triaged to chest pain observation units and undergo provocative testing, at significant cost to the healthcare system. Clinical decision rules use clinical findings (history, physical exam, test results) to suggest a diagnostic or therapeutic course of action. Currently no methodologically robust clinical decision rule identifies patients safe for early discharge.

**Methods/design:**

The goal of this study is to derive a clinical decision rule which will allow emergency physicians to accurately identify patients with chest pain who are safe for early discharge. The study will utilize a prospective cohort design. Standardized clinical variables will be collected on all patients at least 25 years of age complaining of chest pain prior to provocative testing. Variables strongly associated with the composite outcome acute myocardial infarction, revascularization, or death will be further analyzed with multivariable analysis to derive the clinical rule. Specific aims are to: i) apply standardized clinical assessments to patients with chest pain, incorporating results of early cardiac testing; ii) determine the inter-observer reliability of the clinical information; iii) determine the statistical association between the clinical findings and the composite outcome; and iv) use multivariable analysis to derive a highly sensitive clinical decision rule to guide triage decisions.

**Discussion:**

The study will derive a highly sensitive clinical decision rule to identify low risk patients safe for early discharge. This will improve patient care, lower healthcare costs, and enhance flow in our busy and overcrowded emergency departments.

## Background

Patients with acute chest pain often undergo extensive diagnostic testing and risk stratification to diagnose acute coronary syndrome (ACS) and determine the likelihood of future adverse cardiac events. Chest pain can be either cardiac or noncardiac in etiology and represents a continuum of risk from benign self-limiting conditions to life-threatening illness requiring rapid diagnosis and treatment. Currently it is not well established which patients require extensive diagnostic investigation. The goal of this study is to derive a clinical decision rule that predicts adverse cardiac events with a high degree of sensitivity and which will allow emergency physicians to accurately identify patients with chest pain who are safe for early discharge without provocative testing.

### Definition and epidemiology of acute coronary syndromes

ACS is a term that encompasses the disease entities unstable angina pectoris, non-ST-segment elevation myocardial infarction (NSTEMI), and ST-segment elevation myocardial infarction (STEMI). Although myocardial infarction has been defined by a number of clinical, electrocardiographic (ECG), and biochemical characteristics, it is generally agreed that the term indicates death of cardiac myocytes due to prolonged ischemia [1]. Unstable angina pectoris, on the other hand, indicates myocardial ischemia without biochemical evidence of cardiac myocyte death [2].

Data from the 2004 National Hospital Ambulatory Medical Care Survey indicate that chest pain is the second most common chief complaint in North American emergency departments, accounting for 6 million patient visits [3]. Approximately 565,000 patients are ultimately diagnosed with acute myocardial infarction, and nearly twice as many are diagnosed with unstable angina pectoris [4-6].

### Statement of the problem in the emergency department

Chest pain is a diagnostic dilemma for the emergency physician. Data from a recent Canadian study suggest that 4.6% of patients with acute myocardial infarction and 6.4% of patients with unstable angina are misdiagnosed in the emergency department [7], with slightly lower rates reported in the U.S. (2.1% and 2.3%, respectively) [8]. In patients without a prior cardiac history, the challenge is to determine if the chest pain is cardiac in etiology. In patients with a prior cardiac history, the challenge is to determine the short-term risk of adverse outcome.

Information obtained from the history, initial 12-lead ECG, and a single set of cardiac enzymes to detect myocardial necrosis is unable to identify patients who are safe for early discharge with sufficient sensitivity [9,10]. Neither the 2007 ACC/AHA guidelines for the management of patients with unstable angina and NSTEMI nor the practical implementation of the 2002 AHA guidelines for the emergency department proposed by Gibler et. al identify a group of patients at very low risk for adverse cardiac events who can be safely discharged without provocative testing [11,12]. In the absence of guidelines that accurately and reliably identify patients safe for early discharge, physicians' triage decisions are variable and often influenced by level of perceived medical and legal risk [13-15]. As a result patients at very low risk for adverse outcome are often triaged to chest pain observation units and undergo extensive risk stratification protocols based on an unstructured assessment of pretest probability and perceived legal risk [16]. High sensitivity is ensured at the expense of specificity, with increased likelihood of false positive provocative testing and significant cost to the healthcare system.

### Methodologic standards for clinical decision rules

Concomitant with the reporting of various decision rules has been an interest in the methodological standards for their development and validation [17,18]. These standards may be summarized as follows: 1) The outcome or diagnosis to be predicted must be clearly defined and the assessment of this outcome should be made in a blinded fashion. 2) The clinical findings to be used as predictors must be clearly defined and standardized and their assessment must be done without knowledge of the outcome. 3) The reliability or reproducibility of the clinical findings used as predictors must be demonstrated. 4) The subjects in the study should be selected without bias and should represent a wide spectrum of clinical and demographic characteristics to increase the generalizability of the results. 5) The mathematical techniques for deriving the rule must be identified. 6) Clinical decision rules should be sensible: have a clear purpose, be relevant, demonstrate content validity, be concise, and be easy to use in the intended clinical application. 7) The accuracy of the decision rule in classifying patients with (sensitivity) and without (specificity) the targeted outcome should be demonstrated. 8) Prospective validation on a new set of patients is an essential test of accuracy because misclassification is commonly higher when decision rules are tested on a population other than the original derivation set. 9) Implementation to demonstrate the true effect on patient care is the ultimate test of a decision rule; transportability can be tested at this stage.

### Review of previous studies

Currently, there is no decision rule that is widely used in Canadian and U.S. emergency departments. Although a number of studies have been published that risk stratify patients who present to the emergency department with chest pain, none that directly address the clinical question at hand could be considered methodologically robust according to the criteria described previously [19]. Some of the methodologic deficiencies will be described in the following paragraphs.

The specific outcome measures varied considerably among the studies, consisting of acute myocardial infarction alone [20-32], acute myocardial infarction and unstable angina [33-37], acute myocardial infarction and death [38-40], all-cause mortality, acute myocardial infarction, and need for revascularization [10,41-50], and similar composite outcomes with slight variations [19,51-63]. Most studies did not report assessing the outcome without knowledge of the predictor variables.

Fourteen studies reported assessing the predictor variables in a standardized fashion with a data collection sheet specifically designed for a prediction rule study [19,22,23,25,26,31,33,34,47,50,56-59]. However, only four explicitly reported collecting the predictor variables without knowledge of the outcome [19,50,56,57].

Only one study assessed the reliability of the clinical findings to be used as predictors in the rule [19]. However, this study did not report kappa values for the predictor variables considered for inclusion in the rule.

The definition of subjects in previous studies has been extremely variable making it difficult for physicians to interpret and apply the findings to their own patients. Several studies did not specify age criteria for enrolment [23,27-31,33,35,39-41,44,45,48-50,52,55,56,58,60]. Among those that did specify age criteria, different criteria were used: over the age of 18 [21,22,34,38,54,59,63], over the age of 20 [43,46], over the age of 24 [10,24,47], over the age of 25 [19,25,26,53], over the age of 30 [32,36,42,51,61], between 20 and 80 years of age [62], and between 24 and 39 years of age [57,64]. In some studies all patients with a primary complaint of chest pain were eligible for enrolment [19,21,22,24-29,31-34,43,44,46,49,53,54], whereas others required additional or different eligibility criteria [10,23,24,30,35,36,38-42,45,47,48,50-52,54-63,65]. Exclusion criteria varied greatly among the studies as well.

The mathematical techniques were described in all of the studies except one [31]. Several studies developed prediction rules that lacked clinical sensibility and were not easily used in the intended clinical application [21-31,33,34,36,50,52,59,61,62]. Twenty-four studies reported the accuracy of the decision rule in terms of sensitivity and specificity in diagnosing the predicted outcome [10,19,21-30,32-35,45,49,50,54,59,60,62,65].

Twelve prediction rules have been prospectively validated on a different set of patients from which the rule was derived [21,22,25,26,34,37,42,53,55,57,61,65]. None of these have consistently performed with sensitivities of ≥ 98% across studies [66]. Only three prediction rules have been implemented to demonstrate their true effect on patient care [25,36,56]. The clinical decision rule developed by Goldman et al. [25] had a sensitivity of 88% documented in the implementation phase, and the outcome was limited to acute myocardial infarction. Sensitivities as low as 62% have been reported for the decision rule by Selker et al. [36]. Finally, the decision rule developed by Reilly et al. [56] addressed the decision of whether to admit emergency department patients with chest pain to the hospital ward or intensive care unit, not whether to discharge a patient home or arrange additional observation and diagnostic testing.

## Objectives

The goal is to derive a clinical decision rule that is highly sensitive for predicting adverse cardiac events and which will allow emergency department physicians to accurately identify patients with chest pain who are safe for early discharge without prolonged emergency department observation, hospital admission, or provocative testing. Specific objectives are: 1) To develop and pretest standardized clinical assessment methods for patients with acute chest pain, incorporating results of initial cardiac testing. 2) To apply these standardized clinical assessments to patients with chest pain. 3) To determine the interobserver reliability of the clinical findings. 4) To determine the association between the clinical findings and the development of adverse cardiac events within 30 days. 5) To use multivariate techniques to derive a highly sensitive clinical decision rule for patients with chest pain to guide triage decisions and selection of further diagnostic testing. 6) To assess the classification performance of the derived decision rule. 7) To determine emergency physicians' accuracy in predicting acute coronary syndrome without the decision rule.

## Methods/design

### Study design and setting

This will be a prospective cohort study in which consecutive emergency department patients with a chief complaint of chest pain and possible ACS will be enrolled. The study will be conducted a tertiary care academic emergency department in Ottawa, Ontario, Canada with an annual census of approximately 60,000 patient visits.

### Study population

All adult patients at least 25 years of age with a primary complaint of chest pain of at least 5 minutes duration and possible ACS will be eligible for enrolment. Patient eligibility will be determined by the attending emergency physician on duty based on clinical judgment.

Patients will be excluded if any of the following criteria are met: 1) Acute ST-segment elevation (≥ 0.1 mV in limb leads or ≥ 0.2 mV in precordial leads) on the initial ECG. 2) Hemodynamic instability or tachycardia (systolic blood pressure < 90 mmHg, bradycardia < 50 beats/min, tachycardia > 100 beats/min). 3) Pulmonary edema on chest x-ray. 4) Age < 25 years. 5) A history of cocaine use or positive test for cocaine. 6) Severe communication problems such that a reliable history cannot be obtained. 7) A clear traumatic etiology of the chest pain. 8) A radiologically-evident cause of chest pain on chest x-ray (e.g., pneumonia, pneumothorax). 9) Prior enrolment in the study within the past 30 days. 10) Terminal non-cardiac illness. 11) No available phone contact. 12) Pregnancy.

### Patient assessment

All patient assessments will be made by staff physicians who are certified in emergency medicine by the Royal College of Physicians and Surgeons in Canada and/or the College of Family Physicians of Canada. Rotating housestaff will perform patient assessments per standard practice but will be asked to have the staff physicians perform study assessments. The primary investigator will orient each of the physician assessors individually and provide one-on-one training to ensure uniform data collection. All physicians will complete data collection forms after assessing the patient and before obtaining results of diagnostic tests, without knowledge of the outcome.

### Quality assurance

Throughout the duration of the study, the completeness of data collection and compliance in patient enrolment will be monitored. Physicians will be given regular feedback regarding their completeness of data collection. No feedback regarding the reliability or accuracy of each of the predictor variables will be given.

### Selection of variables

The variables selected for assessment in the study were chosen based on review of the literature, input from all the investigators, and solicited feedback from the designator physicians. The number of variables collected was limited to ensure efficient completion of data forms in the context of patient care and optimize physician compliance. The variables to be collected on each patient are listed in Tables 1 and 2.

**Table 1:** List of prospectively collected historical variables.

**Demographics**	• Age (years) • Date of emergency visit (d/m/y)	• Gender (male/female) • Arrival by ambulance
**Cardiac medications**	• Aspirin • Clopidogrel • Other anticoagulants (warfarin, aspirin/dipyridamole) • Beta blockers • Calcium channel blockers	• Nitroglycerin (or other nitrates) • Angiotensin converting enzyme inhibitors • Cholesterol-lowering drugs
**Cardiac risk factors**	• Hypertension • Diabetes Mellitus • Hypercholesterolemia • Renal insufficiency	• Family history of cardiac disease • Smoking history
**Cardiac history**	• Acute myocardial infarction • Cardiac arrest • Peripheral vascular disease • Angina • Ventricular tachycardia	• Known coronary artery disease • Atrial fibrillation • Congestive heart failure • Stroke or transient ischemic attack
**Chest pain characteristics**	• Duration and time of onset of longest episode (days, hours, minutes; a.m., p.m.) • Was the pain present on arrival to the ED? • Is the pain worse with exertion? • Is the pain similar to previously diagnosed ischemia? • Has there been 2 or more episodes of pain in the last 24 hours? • Where on the chest is the pain located? • Does the pain radiate? • Is the pain worse with movement or position? • The physician's overall assessment of the pain (typical or atypical)	• Has the pain completely resolved? • Is the pain present at rest? • Is the pain pleuritic (sharp, worse with deep breathing)? • Has there been a change in the usual pattern of angina within the last 24 hours? • Did the pain recur during the ED visit? • How would you describe the pain? • Is the pain associated with nausea, vomiting, or diaphoresis?

**Table 2:** List of variables to be prospectively collected from the physical examination and diagnostic tests.

	**Variables to be Collected**	
**Physical Examination**	• Temperature (degrees Celsius) • Heart rate (beats per minute) • Systolic blood pressure (mm of Hg) • Diastolic blood pressure (mm of Hg) • Cardiac auscultation findings (S3, S4, Systolic murmur, diastolic murmur)	• Lung auscultation findings (crackles/rales at bases, crackles/rales to scapulae, wheezes) • Chest wall tenderness (reproducing presenting symptom) • Pitting edema in lower extremities
**Diagnostic tests**	• Intepretation of first readable ECG (normal, nonspecific ST-T wave changes, abnormal but not diagnostic of ischemia, infarction or ischemia known to be old, infarction or ischemia not known to be old, consistent with AMI (ST-elevation or new left bundle branch block) • Cardiac stress test done • If yes, type of stress test (nuclear, exercise, stress echo, other) • If yes, result (positive for ischemia, negative for ischemia, equivocal) • If equivocal, mild ischemia, moderate ischemia, or severe ischemia?	• Time and values of first and second cardiac troponin T • Cardiac CT done? • If yes, any stenosis ≥ 70%? • Coronary angiography done? • If yes, any stenosis ≥ 70%? • Did the patient undergo revascularization? • If yes, stent placement, angioplasty alone, or coronary artery bypass grafting?
**Physician judgment**	• Probability of unstable angina or acute myocardial infarction (to the closest percent)	

### Electrocardiogram interpretation and cardiac biomarker assessment

Investigators blinded to the final outcome will review all ECG's in a structured format to identify the presence or absence of ST segment elevation or depression (classified as < 0.05 mV, 0.05 to 0.1 mV, and > 1.0 mV deviation) in at least 2 contiguous leads, T-wave inversion (≥ 0.2 mV when isolated or < 0.2 mV when in 2 or more contiguous leads with dominant R waves), left bundle branch block, right bundle branch block, or pathological Q-waves. Each of these findings will be categorized as "known to be old" or "not known to be old." The overall interpretation of the ECG will be categorized as normal, nonspecific ST-T wave changes, abnormal but not diagnostic of ischemia, infarction or ischemia known to be old, infarction or ischemia not known to be old, or consistent with acute myocardial infarction (ST-segment elevation or new left bundle-branch block). This ECG classification system is known to have high inter-rater reliability and to correlate well with 30-day outcome rates of death, myocardial infarction, and revascularization [67,68].

Cardiac troponin T (cTNT) has been reported to have a higher sensitivity than CK-MB for diagnosis of acute myocardial infarction [69], and current guidelines suggest using cTNT as the sole cardiac marker to detect cardiac ischemia [70]. Thus, the sole cardiac marker utilized in this study will be cardiac cTnT (Elecsys Troponin T, Roche Diagnostics, Indianapolis, Indiana). The 99^th ^percentile of the reference range is < 0.01 μg/L. The lowest concentration at which 10% imprecision is achieved (10% coefficient of variation) is 0.035 μg/L. Some have suggested using the 10% coefficient of variation as the cutoff for myocardial injury to increase specificity and exclude other causes of cTNT elevation such as chronic kidney disease, left ventricular hypertrophy, pulmonary embolism, or sepsis [71,72]. However, several studies have shown that any detectable elevation in cTNT identifies patients at high risk for ischemic complications, and a rising or falling pattern of cTNT can distinguish acute from chronic disease [73-76]. In a robust emergency department trial by Hamm et al. almost every patient at short term risk (30 days) was identified by elevations in cTNT above the 99^th ^percentile [74]. Use of the 99^th ^percentile independent of the coefficient of variation has a very low false positive rate for diagnosing acute myocardial infarction and has recently been validated [77]. Thus, 0.01 μg/L will be the cutoff for a diagnosis of acute myocardial infarction. These reference values conform to the ESC/ACC guidelines for use of existing assays clinically and for clinical trials [2,70].

Having at least a 6 hour interval between cTNT specimens is the AHA definition of an adequate set of biomarkers [2,78]. However, recent data suggest that specimens drawn at least 3 hours apart have the same rate of detection of acute myocardial infarction as the AHA schedule, as long as at least one specimen is drawn ≥ 6 hours after pain onset [79]. Thus, cTNT will be measured at emergency department arrival and ≥ 6 hours from pain onset, with at least 3 hours between samples [79].

### Run-in period

The data collection forms, patient assessment techniques, and patient follow-up questions will be evaluated during an 8-week run-in period prior to the actual study. This will allow time for training of the physician assessors and revision of the data collection forms as appropriate.

### Interobserver reliability

A subset of patients will be assessed by a second emergency physician who will be blinded to the results of the first assessment. These second assessments will be performed on a feasibility basis whenever two study physicians are available.

### Outcome measures

The primary outcome will be acute myocardial infarction, death of cardiac or unknown cause, or revascularization within 30 days of the emergency department visit. The secondary outcome will be acute myocardial infarction, death of cardiac or unknown cause, revascularization, or a new perfusion defect demonstrated on myocardial perfusion imaging.

Acute myocardial infarction will be defined as any one of the following: (1) a cardiac troponin T (cTnT) ≥ 0.01 with a rising or falling pattern (defined as a change of ≥ 0.03 ng/mL for values that are initially <0.2 ng/mL; for levels ≥ 0.20 ng/mL, a positive cTnT will be defined as a change of ≥ 20% between samples)[1,72,80] or (2) development of pathological Q-waves on the ECG or ECG evolution consistent with acute myocardial infarction. Revascularization will be defined as reestablishment of coronary artery patency by percutaneous coronary angioplasty with or without stent placement or coronary artery bypass graft (CABG) surgery. The final component of the primary outcome will be death of cardiac or unknown cause within 30 days of the emergency department visit.

The primary outcome will be determined by investigators blinded to the knowledge of the predictor variables. If a diagnosis cannot be assigned, 2 coinvestigators will review all clinical data and assign an adjudicated outcome diagnosis. If a consensus can not be reached between two co-investigators, an adjudicated diagnosis will be assigned by the primary investigator. If all 3 disagree, the final diagnosis will be the most significant diagnosis. The reliability of the primary outcome determination will be assessed by having all positive outcomes and 10% (randomly selected) of patients with negative outcomes reviewed by an investigator blinded to the first interpretation.

### Data analysis

Interobserver agreement for each variable will be measured by calculating the kappa coefficient, the proportion of potential agreement beyond chance, along with 95% confidence intervals. Variables with kappa values ≥ 0.6 will be considered to represent "substantial agreement" and considered for inclusion in the clinical rule.

### Univariate analysis

Univariate analysis will be used to determine the strength of association between each variable and the primary outcome. The appropriate univariate technique will be chosen for the type of data: for nominal data, the chi-square test with continuity correction; for ordinal variables, the Mann-Whitney U test; and, for continuous variables, the unpaired 2-tailed t-test, using pooled or separate variance estimates as appropriate.

### Multivariable analysis

Multivariable analysis will be used to derive a model to predict the primary outcome. Variables found to be both reliable (kappa ≥ 0.6) and strongly associated with the primary outcome (p < 0.05) will be evaluated with both logistic regression and recursive partitioning. Second order interaction among predictor variables that are known to be clinically related will be evaluated using Mantel-Haenszel and logistic model procedures. Appropriate composite variables will be considered for incorporation in the multivariate analyses. The objective will be to find the best combination of predictor variables that are highly sensitive for detecting the primary outcome while achieving the maximum possible specificity. To be clinically acceptable, the model must be nearly 100% sensitive and contain the fewest number of predictor variables to facilitate ease of use by clinicians.

Recursive partitioning will be performed using KnowledgeSEEKER Version 5.2 software (Angoss Software International, Toronto) [81-83]. In recursive partitioning, the relationship between a dependent outcome variable (Y) and a series of predictor variables (X) is defined by a series of binary splits, resulting in a decision tree in which data are partitioned into several nodes or leaves along branches. The significance of each binary split can be quantified based on the chi-square technique.

Attempts to find the best model will also be made by performing logistic regression as an alternative technique. Model building will proceed with forward stepwise selection until no variables meet the entry (0.05) or removal (0.10) criteria for the significance level of the likelihood ratio test. In order to provide a simpler model for clinicians, cutpoints will be sought for continuous variables. The variables chosen by the best model will constitute the decision rule.

### Classification performance

The derived decision rule will be evaluated by comparing the classification of each patient to their actual status for the primary outcome. This will enable an estimate of the sensitivity and specificity of the rule, with 95% confidence intervals.

### Patient subgroups

The classification performance of the decision rule will be assessed in the following patient subgroups: a) patients with and without a prior cardiac history b) patients with ECG's classified as normal or nonspecific ST-T wave changes and negative cardiac biomarkers and c) patients with outcomes at 0, 4, 14, and 30 days from the emergency department visit.

### Physician judgment

Data from questions relating to physicians' predictions will be tabulated and presented in descriptive format. The probability will be used to calculate a receiving operating characteristic (ROC) curve for the diagnosis of acute coronary syndrome.

### Sample size

Excluding the run-in stage, 1200 patients will be enrolled over 12 months at the study hospitals during phase I. Since no hypothesis is being tested, the sample size is based on estimation of the precision of the sensitivity of the derived decision rule as well as on the precision of the estimates of interobserver variability and the logistic regression coefficients. The sample size has to accommodate a large number of clinical variables (31), a large number of physicians (more than 60), the prevalence of acute coronary syndrome (21% of eligible patients in two recent Canadian studies [7,19]), as well as our plans to assess subgroups. A sample size of 1200 patients with possible ACS in which 11% of cases are excluded for ST segment elevation should yield approximately 120 ACS cases. 120 cases are needed to derive a rule that is 100% sensitive with upper and lower 95% confidence limits of 100% and 97.0%, respectively.

### Ethics approval

Research ethics board approval was obtained from The Ottawa Hospital. As the study will not affect usual practice, there were no specific ethical concerns. At enrolment, participants will be informed that they will be contacted by phone in one month to determine their status, and verbal consent will be obtained at the time of the follow-up phone call. Personal identifiers will be removed from clinical records where present and not stored in the study database.

## Discussion

Chest pain is a diagnostic dilemma for the emergency physician. In the absence of an accurate and reliable method of identifying patients at very low risk for adverse cardiac events, physicians' triage decisions are variable and often influenced by level of perceived medical and legal risk [15]. As a result very low risk patients are triaged to chest pain observation units and undergo extensive risk stratification protocols based on an unstructured assessment of pretest probability and perceived legal risk [16]. Despite this inefficiency, a number of emergency department patients at risk for adverse cardiac events are being missed [8].

We aim to derive a clinical decision rule that is highly sensitive for predicting acute myocardial infarction, need for revascularization, or death within 30 days of presentation to the emergency department using techniques successfully applied to ankle, knee, and cervical spine radiography [84-86]. Future plans are to prospectively validate the derived rule in new set of patients. This will improve patient care, lower healthcare costs, and improve flow in our busy and overcrowded emergency departments.

## Competing interests

The author(s) declare that they have no competing interests.

## Authors' contributions

EH conceived of the study and drafted the manuscript. GW assisted with the statistical design and methodology. AJ assisted with the methodology and revised it critically for important intellectual content. IS assisted with the methodology, revised it critically for important intellectual content, and helped to draft the manuscript. All authors read and approved the final manuscript.

## Pre-publication history

The pre-publication history for this paper can be accessed here:


